# Alcaline Saliva

**Published:** 1844-04-06

**Authors:** 


### ALCALINE SALIVA.

Dr. Wright says, saliva which contains an excess of its natural alcali is always to be regarded as indicative of local or general nervous excitement. This rule may, however, admit of an exception where much carbonate of soda is taken internally.—*Ibid.*

